# ﻿Biodiversity of vertebrates in Argentina: patterns of richness, endemism and conservation status

**DOI:** 10.3897/zookeys.1085.76033

**Published:** 2022-02-04

**Authors:** Valeria Bauni1, Claudio Bertonatti1, Adrián Giacchino1, Facundo Schivo2,14, Ezequiel Mabragaña3,14, Ignacio Roesler4,5,14, Juan José Rosso3,14, Pablo Teta6,14, Jorge D. Williams7,14, Agustín M. Abba8,14, Guillermo H. Cassini8,14, María Berta Cousseau9, David A. Flores10,14, Damián M. Fortunato7, María Emilia Giusti11,14, Jorge Pablo Jayat12, Jorge Liotta13, Sergio Lucero6,14, Tomás Martínez Aguirre7, Javier A. Pereira6,14, Jorge Crisci15

**Affiliations:** 1 Fundación de Historia Natural Félix de Azara. Centro de Ciencias Naturales, Ambientales y Antropológicas, Universidad Maimónides. Hidalgo 775 7mo piso, CP 1405, Ciudad Autónoma de Buenos Aires, Argentina; 2 Instituto de Investigación e Ingeniería Ambiental (IIIA), CONICET-UNSAM, Campus Miguelete, 25 de Mayo y Francia, CP 1650, San Martín, Argentina; 3 Grupo de Biotaxonomía Morfológica y Molecular de Peces (BIMOPE), Instituto de Investigaciones Marinas y Costeras, Facultad de Ciencias Exactas y Naturales, Universidad Nacional de Mar del Plata- CONICET, Deán Funes 3350, CP 7600, Mar del Plata, Argentina; 4 Departamento Científico, Aves Argentinas - Asociación Ornitológica del Plata. Matheu 1246/8, CP 1249, Ciudad Autónoma de Buenos Aires, Argentina; 5 Departamento Análisis de Sistemas Complejos. Fundación Bariloche. EDGE of Existence affiliated. Zoological Society of London, Av. Bustillo 9500, CP 8400, Bariloche, Argentina; 6 División Mastozoología, Museo Argentino de Ciencias Naturales “Bernardino Rivadavia”, Av. Angel Gallardo 470, CP 1405, Ciudad Autónoma de Buenos Aires, Argentina; 7 Facultad de Ciencias Naturales y Museo, Universidad Nacional de La Plata. Anexo Museo, Laboratorio 105. Calles 122 y 60, CP 1900, La Plata, Argentina; 8 Centro de Estudios Parasitológicos y de Vectores (CEPAVE, CONICET-UNLP), Boulevard 120 s/n entre Av. 60 y Calle 64, CP 1900, La Plata, Argentina; 9 Facultad de Ciencias Exactas y Naturales, Universidad Nacional de Mar del Plata, Funes 3550, CP 7602, Mar del Plata, Argentina; 10 Instituto de Vertebrados, Unidad Ejecutora Lillo (CONICET- Fundación Miguel Lillo), Miguel Lillo 251, CP 4000, San Miguel de Tucumán, Argentina; 11 Instituto de Ecología, Genética y Evolución de Buenos Aires (IEGEBA-FCEN-UBA), Ciudad Universitaria, Pabellón II, Güiraldes 2160, CP 1428, Ciudad Autónoma de Buenos Aires, Argentina; 12 Unidad Ejecutora Lillo (CONICET- Fundación Miguel Lillo), Miguel Lillo 251 CP 4000, San Miguel de Tucumán, Argentina; 13 Museo Regional de Ciencias Naturales "A. Scasso", San Nicolás de los Arroyos, Don Bosco 580, CP 2900, Buenos Aires, Argentina; 14 Consejo Nacional de Investigaciones Científicas y Técnicas (CONICET), Argentina; 15 Facultad de Ciencias Naturales y Museo, Universidad Nacional de La Plata. Paseo del Bosque s/n, CP 1900, La Plata, Argentina

**Keywords:** Amphibians, biological inventory, birds, freshwater fish, mammals, marine fish, reptiles

## Abstract

Optimising conservation efforts requires an accurate record of the extant species as well as their geographic distributions. Nevertheless, most current conservation strategies start from an incomplete biodiversity inventory. Argentina has an extraordinary diversity of species, however, until now an updated inventory of its fauna has not been carried out. In this context, the main objective of this work is to present the results of the first national inventory of vertebrate species. Experts from each major vertebrate taxonomic group assembled and compiled its respective inventory. The information gathered included taxonomic rank, conservation status, endemism and geographic distribution. Species richness and representativeness were calculated for each taxonomic group, distinguishing between native, endemic and exotic, for each Argentinian province. Our results show Argentina harbours 3,303 species: 574 marine fish, 561 freshwater fish, 177 amphibians, 450 reptiles, 1,113 birds, and 428 mammals. Native species constitute 98.1% of the total taxa. The results achieved were spatially represented showing a pattern of higher richness from north to south and from east to west. Species considered as threatened account for 17.8% and 15.2% are endemic. There are five Extinct species. These results provide key information on developing strategies and public policies at the national and provincial levels and constitute a tool for the management and conservation of biodiversity.

## ﻿Introduction

There are many estimates of the total number of species in the world, which oscillate by tens of millions (Costello et al. 2012). Nevertheless, most of the world´s biodiversity (as much as 80%) is still entirely unknown thus preventing proper estimates of the total number of species on Earth even to the nearest order of magnitude (Wilson 2003, 2017). The most prudent estimates range from 5 to 50 million species, considering that published species are close to 1.9 million (Chapman 2009). Model-based projections have been performed, indicating that 24–31% marine and 21–29% terrestrial species remain to be discovered (Costello et al. 2012). The Catalogue of Life, which contains contributions from 172 taxonomic databases, estimates 2,260,074 species accepted or provisionally accepted in 2020 (Roskov et al. 2020). In 2019, 59,284 species were estimated to have become extinct before and during the Holocene (Roskov et al. 2019). Additionally, it has been estimated that human activities have already led to the extinction of at least 680 species of vertebrates since 1500 (IPBES 2019).

Recently, the IPBES Panel (Intergovernmental Science-Policy Platform on Biodiversity and Ecosystem Services) drew the world’s attention by confirming that human actions have raised -and accelerated- the global extinction rate of wild species at an unprecedented rate when compared to the last 10 million years. So much so that 25% of animals and plants species assessed by the International Union for Conservation of Nature (IUCN) are threatened (IPBES 2019).

In this context, optimising conservation efforts requires accurately recording species and assessing where they live (Costello et al. 2013). Regrettably, current conservation efforts usually start from incomplete biodiversity catalogues (Scheffers et al. 2012). An inventory lists, orders, catalogues, and quantifies ecoregions, ecosystems, and/or species (Stork and Samways 1995, PNUD 2007). Inventorying is a fundamental tool for environmental management (McNeely et al. 1995) as what is unknown cannot be protected. Therefore, it constitutes the first and most reasonable conservation action (Evenhuis 2007). Since species are the fundamental units of biology, ecology, and conservation assessments (Mace 2004; Tobias et al. 2010; Costello et al. 2013), most biological inventories are presented at this level of biological hierarchy.

The earliest systematic record of biodiversity in Argentina dates back to the studies of Félix de Azara (Azara 1801, 1802–1805). Since then, lists, catalogues, and reference collections have been added, which require being constantly updated. In Argentina, extraordinary ecosystem diversity results in a great diversity of species. In the case of faunal species, precise estimates of their richness are mostly scattered and outdated. For the case of plant species, there is an updated and complete national catalogue comprising 10,221 species of vascular plants (Zuloaga et al. 2019). According to the IUCN (2021), there are about 320 threatened species at the global scale, including vertebrates, invertebrates, plants, and fungi present in Argentina.

Amidst a global change crisis, knowing the list of existing taxa became essential (Scheffers et al. 2012), especially for different political jurisdictions, including their systematic identification, their geographical distribution and their conservation status. In most countries of the world, this knowledge is fragmentary, incomplete, and outdated. This aspect becomes particularly complex in a context in which global wildlife populations are evidently declining, yet simultaneously, new taxa continue to be described (Costello et al. 2013; Grismado and Ramírez 2018, 2019, 2020).

Despite representing only 3.45% of described species (73,118 species) and a much lower fraction of extant species (IUCN 2021a), vertebrates have been used to make extrapolations in a wide range of biodiversity and conservation analyses (Titley et al. 2017; Fukushima et al. 2020). Particularly in Argentina, there is a lack of a single, complete, and updated inventory of vertebrate fauna at the national or provincial level. Having an inventory of national scale is particularly timely in a context dominated by a widespread land use and land cover change intensification, accompanied by a gradual degradation and destruction of natural communities. Completing an inventory of known species at the country level is therefore a priority for both biodiversity data management and conservation (Costello et al. 2012). In this context, the main objective of our work is to analyse the results of Argentina’s first national inventory of vertebrates under the premise that developing objective decision-making and establishing precise public policies demands this type of information (Webb et al. 2010; Costello et al. 2013). As a consequence, the main objective of this collective effort is to be kept up-to-date and free for decision-makers.

## ﻿Material and methods

### ﻿Study area

**Figure 1.**
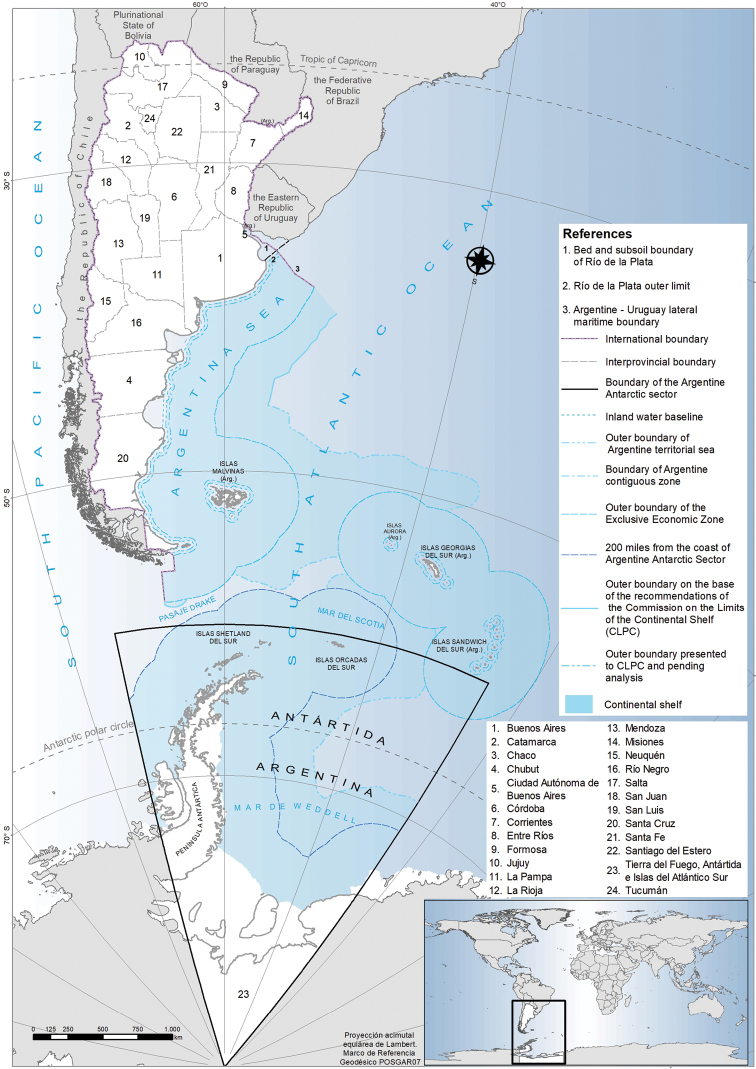
Political map of Argentina. International and national boundaries, including terrestrial and maritime, are indicated. Each of the 23 provinces and the autonomous city of Buenos Aires are depicted. Source of spatial information: National Geographic Institute (IGN 2021).

The continental area of Argentina extends for 2,791,810 km² (IGN 2019), which makes it the second largest country in South America after Brazil, and the eighth largest in the world, considering its continental area subject to effective sovereignty (Arana et al. 2021). It covers a large part of the Southern Cone of South America, bordered to the north by Bolivia and Paraguay, to the northeast by Brazil, to the east by Uruguay and the Atlantic Ocean, to the west by Chile, and to the south by Chile and the waters of the Drake Passage (Fig. 1; Arana et al. 2021). Latitudinally, it is an extensive country, ranging from 21°45'S (at its northern limit) to 53°03'S (at its southernmost part). A mountainous range extends along the western edge with peaks exceeding 7,000 metres above sea level. A third of its territory is semi-arid, arid and desert (Morello et al. 2012). A wide diversity of climates is present, from tropical and subtropical in the northwest and northeast, to extreme cold in the mountain zones and the south. The most extensive climate is temperate. As a consequence of its vast territory, it exhibits a great diversity of biomes, from salt flats and deserts, temperate forests to subtropical forests, shrublands, grasslands and wetlands (Arana et al. 2021). The coast covers a distance of 4,645 km (Acha 2014). Morello et al. 2018 identified 16 ecoregions in Argentina, including the Argentinian Sea (Mar Argentino). Argentina’s territorial organisation is made up of several levels. It comprises 23 provinces and the autonomous city of Buenos Aires, which is the capital of the nation. Argentina extends its sovereignty over the sea adjacent to its coasts and islands, as well as over the bed and subsoil of marine areas that cover 1,785,000 km^2^ (Fig. 1; Acha 2014; Gaitan 2020). Tierra del Fuego, Antártida e Islas del Atlántico Sur Province includes territories whose sovereignty is in dispute: Islas Malvinas (Malvinas/Falkland Islands), Islas Georgias del Sur (South Georgia Islands), Islas Sandwich del Sur (South Sandwich Islands), Islas Orcadas del Sur (South Orkney Islands), Islas Shetland del Sur (South Shetland Islands), Islas Aurora (Aurora Islands), and Antártida Argentina (Argentina Antarctic Sector).

### ﻿Database generation

Experts were convened to elaborate and compile an updated inventory of vertebrate species in Argentina: marine and freshwater fishes, amphibians, reptiles, birds, and mammals. In order to expedite the following analyses, a single merged database was compiled for all taxa, which included the following information for each recorded species: Class, Order, Family, scientific name, common name, synonyms, and national conservation status (or international, in the case of groups that did not have national evaluations; e.g., marine fish). If a species was endemic to Argentina, the region of endemism and distribution (presence by province) were also included. Argentinian provinces have authority over their natural resources and conservation actions must be conducted in agreement with the corresponding authorities. Therefore, the presentation of results segregated by provinces is not a matter of convenience, but applicability. The inventory also considers introduced, invasive and/or exotic species.

The conservation categories used by the different national lists were homologised to unify criteria differing between them, and fit to the international categories of the IUCN (Table 1). Species classified as Critically Endangered (CR), Endangered (EN) or Vulnerable (VU) were considered threatened (Gärdenfors 2001; IUCN 2019). The “Regionally Extinct” category was incorporated, and was used for those species that are extinct within, for example, a particular country but that are still extant in other parts of the world (Gärdenfors 2001).

**Table 1.** Conservation categories applied for Argentina´s vertebrate inventory.

Unified Conservation Status Categories	Acronym
Extinct	EX
Extinct in the Wild	EW
Regionally Extinct	EXR
Critically Endangered	CR
Endangered	EN
Vulnerable	VU
Near Threatened	NT
Least Concern	LC
Not Threatened	NA
Data Deficient	DD
Not Evaluated	NE
Not applicable	NAP

**Marine fishes.** The list of marine fish compiles information that includes the continental shelf and slope between 34°S and 55°S and the Uruguayan shelf based on the existence of the Argentina-Uruguay Common Fishing Zone. It is based on different bibliographic sources (Pozzi and Bordalé 1935; Menni et al. 1984; Cousseau et al. 2010; Cousseau and Rosso 2019; Figueroa 2019) as well as research conducted by the National Institute for Fisheries Research and Development (Instituto Nacional de Investigación y Desarrollo Pesquero, INIDEP) and the Puerto Deseado Oceanographic Vessel. Contributions made by commercial and sport fishermen were also included, since they report their catches to INIDEP (Cousseau et al. 2010). Both valid scientific names and known synonyms of fish species were assigned according to Fricke et al. (2020). For suprageneric categories, Nelson et al. (2016) was followed. Regarding endemics, those reported for the Magellan Province were included (Cousseau et al. 2020). With respect to the geographical distribution of each species, the information available worldwide has been considered, since most species exceed the limits of the Argentinian continental shelf. Conservation status corresponds to that assigned by the IUCN, since no national categorisation exists.

**Freshwater fishes.** The list was compiled from different information sources regarding the presence and distribution of freshwater fish in Argentina (Ringuelet et al. 1967; López et al. 1987, 2003; Menni 2004; Liotta 2005; Mirande and Koerber 2015, 2020; Cousseau and Rosso 2019, in press) and the database fish from continental water (Base de Datos de peces de Agua Continentales de Argentina). This Inventory includes some species not considered in previous publications. For systematic information, we followed Nelson et al. (2016) and for the synonymy, Fricke et al. (2020). Conservation aspects have been incorporated considering all currently available works, which have variously conducted evaluations at the national, regional or local level (Chebez 1994; Bello and Ubeda 1998; Orlandini et al. 2001; López et al. 2003; Cordiviola and Zayas 2007; Cappato and Yanosky 2009; Chebez et al. 2009; Cordiviola et al. 2009; Alonso et al. 2018; Cardoso et al. 2019). When a species was placed in different conservation categories according to the various information sources consulted, we kept the highest degree of threat, as a precautionary principle (Bauni et al. 2021). Some exceptions were made for very restricted regional or local evaluations of some species where the highest category did not accurately represent the national scenario for the species.

**Amphibians and reptiles.** For the compilation of these groups the information was obtained from an exhaustive bibliographic review, comprising lists published by Avila et al. (2013) for lizards and amphisbaenians; Williams and Francini (1991), Giraudo and Scrocchi (2002), and Williams et al. (2021) for snakes; the conservation categorisations published by the Argentina Herpetological Association (AHA, Spanish abbreviation) in 2000 and 2012. Also, different regional field guides were consulted, including digital databases such as “Amphibian Species of the World” (Frost 2021) for amphibians and “The Reptile Database” (Uetz 2021) for reptiles. For the conservation status the last proposal generated by the AHA was followed (Abdala et al. 2012; Giraudo et al. 2012; Prado et al. 2012; Vaira et al. 2012).

**Birds.** Taxonomic order was based on the combination of different sources frequently used by Neotropical ornithologists, which are mostly used as references in scientific publications from Argentina (e.g., El Hornero and Nuestras aves). Systematics follows the nomenclature proposed by specialists in the “Argentina Committee of Ornithological Records” (CARO, Spanish abbreviation) (Monteleone et al. 2021) and that proposed by the South American Classification Committee (SACC) (Remsen et al. 2021). However, modifications were made following some extra sources of popular use, such as eBird. In the same way, some updates were made following BirdLife International (2021). To generate Argentina’s bird database, the lists of Monteleone and Pagano (in prep.) and Pearman and Areta (2018) were used as the main sources. Field guides were used for provincial distribution (Fjeldså and Krabbe 1990; Rodríguez Mata et al. 2006; Ridgely and Tudor 2009; Narosky and Yzurieta 2010; Pearman and Areta 2018, 2020) as were regional or provincial guides and publications (Nores et al. 1991; Narosky and Giacomo 1993; De La Peña 1997). In order to provide updated information at the provincial level, databases such as eBird were also consulted (eBird 2021), as well as periodic national publications (e.g., Nuestras aves, Nótulas Faunísticas, Cotinga). Areas of endemism were mainly based on Mazar et al. (2001) and Pearman and Areta (2020) with modifications based on empirical observations and modern literature. Species of hypothetical historical presence were not considered. The species conservation status was based on the last national categorisation (López-Lanús et al. 2017), except for species not yet considered in that list. In those cases, Birdlife was consulted (BirdLife International 2021).

**Mammals.** The taxonomic list in this work was based on Teta et al. (2018), with modifications according to more recent literature. The aforementioned list includes living species and those considered extinct or potentially extinct in Argentina during historical times (i.e., since 1500 AD). It excludes species of hypothetical or probable presence in the country. In the case of exotic species, only those taxa with one or more recently documented wild populations are considered (Chebez and Rodríguez 2014; Teta et al. 2018). For the conservation status of this group, the last national categorisation was used (SAyDS and SAREM 2019).

### ﻿Data compilation and analyses

The complete list of all vertebrates was published as a book and is freely accessible at the following web: https://www.fundacionazara.org.ar/img/libros/inventario-biologico-argentino.pdf (Bauni et al. 2021). For each province, species richness and percentage of representativeness were calculated for each taxonomic group, distinguishing between native, exotic, endemic, and threatened taxa. For species representativeness, the total of each category at the national level was considered. The number of exclusive endemic species per province for each group was also evaluated. The results achieved were spatially represented through the elaboration of cartographic products. For each province, we used a colour gradient to depict species richness values. For visualisation, only the continental area of the American continent was mapped (Antarctica was excluded). Marine species were assigned to Argentinian Sea as a whole unit for map representation, but it does not necessarily mean that the species inhabit the entire region. The same criteria were used for Tierra del Fuego, Antártida e Islas del Atlántico Sur, thus the use of the full name does not imply that the species is present throughout that territory.

## ﻿Results

Argentina’s national vertebrate inventory comprises 3,303 species: 574 marine fish, 561 freshwater fish, 177 amphibians, 450 reptiles, 1113 birds and 428 mammals. In total, 98.1% are native (3,240 spp.) and 15.2% (492 spp.) endemic (Table 2). The taxonomic groups with the highest number of introduced, invasive, and/or exotic species are freshwater fish (22 spp.), and mammals (21 spp.). The latter has the highest percentage (4.9%) regarding the total species of its group.

**Table 2.** Total number (and percentage) of species richness, native species, exotic species, and percentage endemism by taxonomic group. *The percentage of endemic species is calculated over the total of native species of the group.

Taxonomic group	Total	Native	Exotic	Endemic*
Marine fishes	574 (17.4%)	570 (99.3%)	4 (0.7%)	20 (3.5%)
Freshwater fishes	561 (17%)	539 (96.1%)	22 (3.9%)	96 (17.8%)
Amphibians	177 (5.4%)	176 (99.4%)	1 (0.6%)	52 (29.5%)
Reptiles	450 (13.6%)	446 (99.1%)	4 (0.9%)	216 (48.4%)
Birds	1,113 (33.7%)	1,102 (99.0%)	11 (1.0%)	21 (1.9%)
Mammals	428 (13%)	407 (95.1%)	21 (4.9%)	87 (21.4%)
Total	3,303 (100%)	3,240 (98.1%)	63 (1.9%)	492 (15.2%)

**Figure 2.**
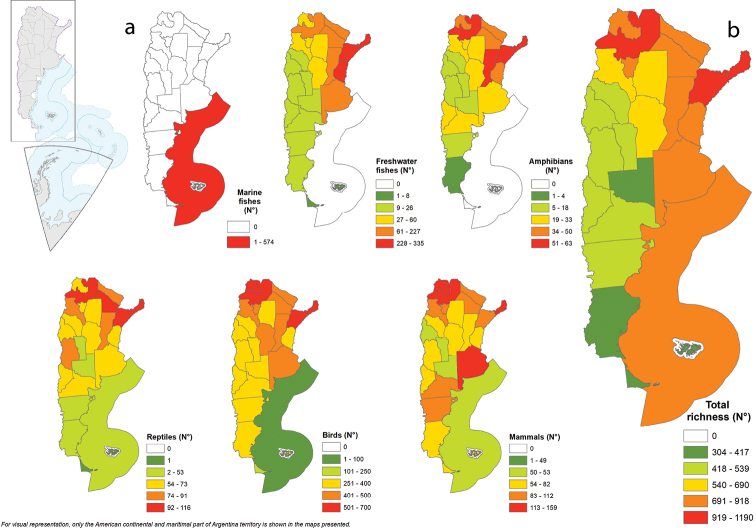
Species richness **a** by taxonomic group by province and **b** total species richness.

Misiones province exhibits the highest species richness of continental vertebrates in Argentina (1,190 spp.) followed by Salta (1,092 spp.) and Corrientes (1,079 spp., Fig. 2, Appendix 1: Table A1–A3). Misiones also has the highest richness of freshwater fish species (335 spp.) and amphibians (63 spp.), whereas Salta has the largest number of species of native reptiles (116 spp.), birds (603 spp.) and mammals (159 spp.) (Fig. 2, Appendix 1: Table A1–A3). The lowest number of species (304 spp.) is observed in Tierra del Fuego, followed by Santa Cruz (382 spp.) (Fig. 2, Appendix 1: Table A1–A3).

Neuquén has the highest number of exotic species, which includes five freshwater fishes and five birds as well as eleven mammals. Santa Cruz has the highest percentage of exotic freshwater fishes (six species, 46.2%; Appendix 1: Table A1–A3).

**Figure 3.**
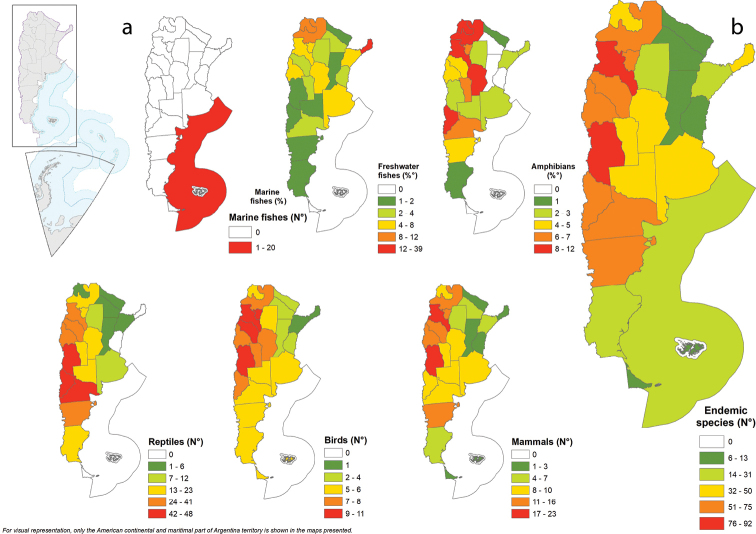
Number of endemics **a** by group by province **b** total species by province.

Catamarca displays the highest number of endemic species (41 reptiles, 23 mammals, nine amphibians, and eight freshwater fishes) (Fig. 3, Appendix 1: Table A1–A3). Misiones has the highest number of endemic freshwater fishes (39 spp.), Jujuy the highest number of endemic amphibians (12 spp.), Neuquén of reptiles (48 spp.), followed by Mendoza and Río Negro (47 spp. each) and Catamarca of birds (11 spp.) and mammals (23 spp.; Fig. 3, Appendix 1: Table A1–A3). Neuquén is the province with the highest proportion of endemic vertebrate species (17.6%). In particular, reptiles comprise 70% of endemic species in this province. There are 321 endemic species exclusive of some provinces of Argentina (Table A2). Misiones has the largest number of exclusive endemics (38 spp.), with 35 species of freshwater fish, two amphibians, and one mammal. Neuquén has 33 exclusive endemic species, with 26 exclusive species of reptiles, six amphibians and one mammal. Catamarca has 31 exclusive endemic species to the province, including 17 reptiles, five freshwater fish and mammals, and four amphibians (Appendix 2: Table A4).

Species considered as threatened (577 spp.) account for 17.8% of all native species, comprising 198 birds, 133 reptiles, 98 mammals, 74 marine fishes, 27 freshwater fishes, and 47 amphibians (Table 3). Marine fishes under threat represent 13.0%, although none of the 20 endemic species is under threat. Five percent of native species of freshwater fish are under threat and 36% of species are in the Near Threatened category. Endemic freshwater fish under threat represent 11.5% of species. Of amphibians 26.7% of all species under threat and 63.5% of endemic species are threatened. Eighteen percent of reptiles are in threatened categories and 25.9% of endemic species are under threat (Table 3). There are two extinct birds (*Numeniusborealis* and *Anodorhynchusglaucus*) and three are categorised as possibly Regionally Extinct (*Taoniscusnanus*, *Primoliusmaracana* and *chloropterus*). There are 198 birds in threatened categories and 57.1% of endemic species are threatened. There are 98 mammals under threatened categories: three are listed as Extinct (*Dusicyonaustralis*, *Dusicyonavus* and *Gyldenstolpiafronto*) and two as Regionally Extinct (*Monodelphisunistriata* and *Pteronurabrasiliensis*). A total of 32 endemic mammals is threatened (36.8%).

**Table 3.** Number of species in each conservation status category and total numbers and percentages of threatened and threatened endemic species (EX, Extinct; EXR, Regionally Extinct; CR, Critically Endangered; EN, Endangered; VU, Vulnerable; NT, Near Threatened; LC, Least Concern; NA, Not Threatened; DD, Data Deficient; NE, Not Evaluated; NAP, Not Applicable; “?”, possible). *CR, EN, VU, percentages are calculated over the total of native species of the group. ** Percentages are calculated over the total of endemic species of the group.

Taxonomic Group	EX	EXR	EXR?	CR	EN	VU	NT	LC	NA	DD	NE	NAP	Threatened species*	Threatened Endemic species**
Marine fishes	–	–	–	17	17	40	16	300	–	35	143	2	74 (13.0%)	0 (0.0%)
Freshwater fishes	–	–	–	3	2	22	194	115	12	31	160	–	27 (5.0%)	11 (11.5%)
Amphibians	–	–	–	–	18	29	–	–	100	20	9	–	47 (26.7%)	33 (63.5%)
Reptiles	–	–	–	–	38	95	–	–	218	49	46	–	133 (29.8%)	56 (25.9%)
Birds	2	–	3	18	90	90	–	790	–	23	86	–	198 (18.0%)	12 (57.1%)
Mammals	3	2	–	7	26	65	40	175	–	72	6	11	98 (24.1%)	32 (36.8%)
Total	**5**	**2**	**3**	**45**	**191**	**341**	**250**	**1380**	**330**	**230**	**450**	**13**	**577 (17.8%)**	**144 (29.3%)**

Twenty-one percent of species were Not Evaluated or Data Deficient, with fish contributing the largest number of species (191 freshwaters, 178 marines).

**Figure 4.**
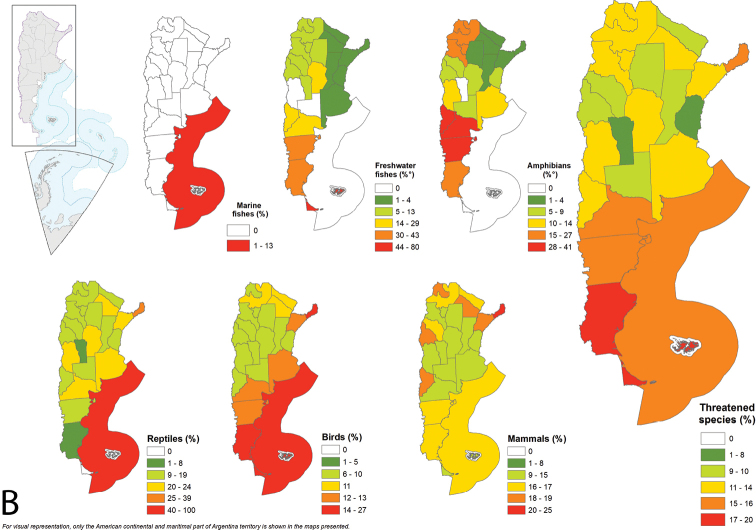
Threatened species by taxonomic group and province **A** number of threatened species by taxonomic group and total number of total threatened vertebrate species in each province **B** percentage of threatened species over the number of total native species of each taxonomic group present in the province and total threatened species in each province as a percentage of total vertebrate species.

Misiones has the highest number of threatened vertebrate species (CR, EN, VU) with 176, which corresponds to 15% of extant native species in the province. The total number of threatened species is higher in northern provinces and in the Argentinian Sea (Fig. 4A), while the percentage of threatened species is higher in southern provinces, except for Misiones (Fig. 4B). In Tierra del Fuego, 80% of freshwater fish are under threat. In Chubut, 41.2% of amphibians present are in danger. Almost 40% of reptiles and 23.7% of extant mammals in Misiones are threatened. In the Argentinian Sea, 100% of present reptiles (e.g., marine turtles) and 26.6% of extant birds are under threat (Fig. 4A, B, Appendix 3: Table A5).

## ﻿Discussion

The results obtained in this study constitute the first analysis of geographical occurrence and conservation status, which highlights endemism, of all vertebrates that inhabit Argentina. Moreover, results are further disaggregated by both native and exotic species. Altogether, this study represents a precise, updated and spatially explicit source of information of vertebrate species, at both the national and provincial levels, for all assessed taxonomic groups. In this regard, it may serve as a reliable tool for multiple uses and users. The information generated by experts in this study establish the foundations for further research in multiple aspects and disciplines of conservation science, involving the assessed taxa. Our results facilitate prioritising research lines and conservation programmes in-situ and ex-situ, further assisting researchers and decision-makers focusing on either endemic or threatened species. In addition, we expect our products to become essential for local decision-makers, who usually lack spatially explicit information regarding actual biodiversity in their areas. This inventory might also be used as background information to update legislation in order to strengthen the protection of endemic and endangered species in each province. More importantly, it will provide key assistance in clarifying the potential geographic distribution of species captured, hunted, traded, or illegally introduced into the country.

The National Biodiversity Strategies and Action Plan (NBSAP) is a process by which countries can plan to address the threats to their flora and fauna. They are the principal instruments for the implementation of the Convention on Biological Diversity, both at the national and at the global level (Secretariat of the Convention on Biological Diversity 2011). Since the NBSAP should be a dynamic process by which increasing scientific information and knowledge must be considered as relevant feedback for a permanent review process, the results of this research should be considered in Argentinian strategies. Additionally, neighbouring countries, which share many of the assessed vertebrates species, could find valuable data in this inventory.

Updating inventories of species is a continuous and tedious process, as new descriptions and nomenclatural changes are published. One of the most complex tasks to complete in this study was to collect information, from different sources such as systematic lists or databases, field surveys, bibliographic reviews and analysis of natural history collections. Simultaneously, taxonomic changes may occur while collecting information. Another complex challenge was introduced by non-standardised and differing conservation categories. The differing national catalogues for each taxonomic group, when present, use different criteria in their classifications. To even these differences, this work unifies the aforementioned criteria with the international categories in order to comprehensively analyse data and make worldwide comparisons, when applicable. Marine fishes do not have national categorisation, and the IUCN Red List criteria were applied to assess their extinction risk at the global level. Using these criteria on a national scale poses disadvantages (Gärdenfors 2001) and reveals the importance of being able to categorise all groups based on their current status at the national level.

Latin America and the Caribbean region support rich biological diversity, accounting for around 60% of global terrestrial life, alongside with diverse freshwater and marine flora and fauna (UNEP and WCMC 2016). In Latin America, it is estimated that there are at least 13,600 vertebrate species (Raven et al. 2020). When considering Argentina’s neighbouring countries, Brazil, one of the largest countries in the world, exhibits the greatest richness of vertebrate species: 8,930 in 8,516 million km² (ICMBio 2021). Bolivia, which has one of the most diverse vertebrate faunas in the world, has registered 3,329 species (MMAyA 2018) in an area of 1,099 million km². Our results allow us to postulate that the vertebrate richness of Argentina is close to the values reported for Bolivia, with 3,302 reported species. Chile has an incomplete faunal inventory (it is estimated that only 10% has been surveyed) with approximately 2,000 vertebrates verified in a total area of 756,950 km² (Ministerio del Medio Ambiente 2021). In Paraguay, there is an estimated richness of 1,500 vertebrates, although a complete inventory of vertebrate species that inhabit its territory (406,752 km²) is still lacking (Maceo et al. 2015). Finally, Uruguay harbours 912 species of vertebrates (without considering marine fishes) in 176,215 km² (Soutullo et al. 2013; Achaval 2021).

The decline in species richness as latitude increases is one of the most consistent patterns in biogeography, having been identified in groups of organisms such as mammals, fish, insects, and plants (Willig et al. 2003). Argentina shows a pattern of higher richness from north to south and from east to west (Fig. 2b), where Misiones and Salta have the highest number of species and Tierra del Fuego and Santa Cruz, the southernmost provinces, are those with the lowest vertebrate richness. This pattern is consistent with the findings of other researchers who have documented that at the Neotropical/Andean level (Morrone 2015) species richness of terrestrial vertebrates is lower on the west coast and in southern South America (Loyola et al. 2009).

Almost 18% of vertebrate species present in Argentina are threatened. The taxonomic group with the highest number of threatened is reptiles, with almost 30% of their species under some category of threat. On the other hand, amphibians have 63.5% of endemic species under threat. Argentina has five Extinct species, two Regionally Extinct and three possibly Regionally Extinct, belonging to mammals and birds. Among mammals, *Pteronurabrasiliensis* has not been recorded in the country since 1980 but a solitary specimen has recently been observed in Chaco and Formosa provinces. Among birds, the extinct *Primoliusmaracana* was last recorded in the 1990’s (Bodrati et al. 2006) and *Paraclaravisgeoffroyi*, a Critically Endangered species, is possibly Extinct (Lees et al. 2021). Richness patterns for threatened and endemic species do not show a relationship to latitude and differed in terms of overall richness, which differ substantially among taxa, as observed at the Neotropical/Andean and global scale (Loyola et al. 2009; Jenkins et al. 2013). The highest number of threatened freshwater fishes is concentrated in Corrientes, Entre Ríos, Buenos Aires, Santa Fe and Salta (Fig. 4A). A higher number of threatened amphibians occur in the northwest provinces Jujuy and Salta (Fig. 4A). Threatened mammals and reptiles are concentrated in northern provinces as well (Misiones, Formosa, Chaco, Salta and Jujuy; Fig. 4A). In contrast, threatened birds are scattered throughout the country. Tierra del Fuego, the southernmost province, exhibits the largest proportion of threatened species considering the species that inhabit it (19.2%, Fig. 4B). This might be related to different drivers that cause species declines. For terrestrial and freshwater ecosystems, land-use change has had the largest negative impact on nature, followed by the direct exploitation of organisms. In marine ecosystems, the exploitation of organisms (mainly fishing) has had the largest impact. Climate change is a driver that is increasingly exacerbating the impact of other drivers on nature (Allan et al. 2019; IPBES 2019). Because of its great diversity of environments, Argentina has a wide range of threats and pressures on its ecosystems. Anthropogenic pressures associated with land use, mostly in terrestrial ecoregions, are livestock grazing and agriculture. However, land use intensification is not homogeneous throughout the country. Different human-activities and processes stress biodiversity based on the characteristics of each ecoregion, such as biological invasions, urbanisation, subsistence livestock, afforestation, the extraction of natural resources, and hunting, among others (Nanni et al. 2020).

Worldwide, 27% of mammals, birds, reptiles, and amphibians are threatened by invasive alien species (Bellard et al. 2016). In this present research, 35% of reported exotic species are freshwater fish and 33% are mammals. Globally, invasive alien species are not the most important contributor to the number of species that are threatened (Bellard et al. 2016), still biological invasions are one of the principal drivers of biodiversity loss (IPBES 2019).

Argentina has 492 endemic vertebrate species, which represent almost 15% of the native vertebrates of the country. Approximately, 50% of reptiles and 30% of amphibians are endemic. This information is valuable for planning conservation strategies. Apart from threatened species, endemic species are indeed an important target of global conservation efforts (Loyola et al. 2009; Murali et al. 2021) since they have a restricted geographical distribution and are more vulnerable to habitat loss or degradation (Prendergast et al. 1993). Our assessment revealed that most endemic species occur in north-western forested areas (Southern Andean Yungas) or in arid to semiarid environments of central, southern, and western Argentina (High and Low Monte and Patagonian Steppe). These results agree with previously performed studies of global phylogenetic endemism patterns for vertebrates (Murali et al. 2021). In this matter, endemism increases southward, peaking at high latitudes in the Southern Hemisphere and coastal areas adjacent to mountain systems (e.g., along the Andes).

If we consider the species in Not Evaluated and Data Deficient categories altogether, they totalise 21% of the total vertebrate diversity of Argentina. Freshwater and marine fish are taxonomic groups with the highest number of Not Evaluated species (35.4% and 31.2%, respectively). This number is higher than threatened species and shows that these species should be regarded as relatively high priorities for research in order to clarify their true status (Butchart and Bird 2010). Birds are the most completely assessed taxonomic group regarding conservation status, with only 10% of the species under the Not Evaluated or Data Deficient categories.

Protected areas (PA) are critical for biodiversity conservation (Saura et al. 2018). The fate of many endangered species depends on PA systems that must be well designed and properly managed (Saura et al. 2017). Nevertheless, the protected area system at the national level in Argentina represents 13.3% (SIFAP 2020), which is still insufficient. Furthermore, the number of protected areas and their included spatial extent are not homogeneously distributed among provinces (SIFAP 2020). Although strongly increased in recent years, Marine Protected Areas represent only ~ 7% of the Argentina Sea (SIFAP 2020), which is still far from the 10% conservation goal set for 2020 in the Convention on Biological Diversity 2010. We believe the information obtained in this research identifies provinces with a particularly high number of threatened or endemic species. Linking this information with the degree of protection at each political district allows the identification of provinces where prioritising the creation of PA is necessary, either by the State, non-governmental organisations or private owners.

## ﻿Conclusions

The importance of compiling a national inventory of vertebrate species is not only relevant from a taxonomic standpoint. It also constitutes a mandatory input in further assessing current biodiversity, as well as in prioritising efforts in environmental management, decision-making, and development of public policies at the national or provincial level. For instance, identifying priority provinces or taxa for *in situ* or *ex situ* conservation, science and education, and developing monitoring and early warning systems in the presence of exotic species that can potentially become invasive. This inventory provides the basis to analyse, study, objectively quantify, monitor, prioritise and value the vertebrate biodiversity of Argentina. In addition, to update the legislation, document the current diversity and geographic occurrence of species (as a future reference) and provide citizens with a simple tool that allows them to know their natural heritage.

Only results for a single animal subphylum are presented here. In the future, the final objective of our initiative is to include groups of invertebrates, which represent a larger volume of species. When completed, Argentina will have a complete national inventory of animal biodiversity. The effort at this scale should stimulate a continuity that emulates the Catalogue of Life (Roskov et al. 2019) or the Encyclopedia of Life (Parr et al. 2014) at the national and provincial levels.

## ﻿Appendix 1

Total number (and percentage, regarding national richness) of native and endemic species by taxonomic group and province. The number of exotic species is the difference between the richness and the number of native species in each case. * Buenos Aires includes Ciudad Autónoma de Buenos Aires. ** Not all species are endemic to the Argentina Sea but may be from a portion of it.

**Table A1.**

Taxonomic group	Marine fishes				Freshwater fishes				Amphibians			
Province	Richness	Native	Exotic	Endemic	Richness	Native	Exotic	Endemic	Richness	Native	Exotic	Endemic
Buenos Aires*	0 (0.0%)	0 (0.0%)	0 (0.0%)	0 (0.0%)	227 (40.5%)	216 (40.1%)	11 (50.0%)	6 (6.3%)	30 (16.9%)	29 (16.5%)	1 (100.0%)	2 (3.8%)
Catamarca	0 (0.0%)	0 (0.0%)	0 (0.0%)	0 (0.0%)	38 (6.8%)	35 (6.5%)	3 (13.6%)	8 (8.3%)	26 (14.7%)	26 (14.8%)	0 (0.0%)	9 (17.3%)
Chaco	0 (0.0%)	0 (0.0%)	0 (0.0%)	0 (0.0%)	166 (29.6%)	165 (30.6%)	1 (4.5%)	4 (4.2%)	48 (27.1%)	48 (27.3%)	0 (0.0%)	0 (0.0%)
Chubut	0 (0.0%)	0 (0.0%)	0 (0.0%)	0 (0.0%)	17 (3.0%)	12 (2.2%)	5 (22.7%)	2 (2.1%)	17 (9.6%)	17 (9.7%)	0 (0.0%)	4 (7.7%)
Córdoba	0 (0.0%)	0 (0.0%)	0 (0.0%)	0 (0.0%)	56 (10.0%)	50 (9.3%)	6 (27.3%)	7 (7.3%)	33 (18.6%)	32 (18.2%)	1 (100.0%)	10 (19.2%)
Corrientes	0 (0.0%)	0 (0.0%)	0 (0.0%)	0 (0.0%)	297 (52.9%)	296 (54.9%)	1 (4.5%)	8 (8.3%)	59 (33.3%)	59 (33.5%)	0 (0.0%)	2 (3.8%)
Entre Ríos	0 (0.0%)	0 (0.0%)	0 (0.0%)	0 (0.0%)	265 (47.2%)	259 (48.1%)	4 (18.2%)	4 (4.2%)	42 (23.7%)	42 (23.9%)	0 (0.0%)	0 (0.0%)
Formosa	0 (0.0%)	0 (0.0%)	0 (0.0%)	0 (0.0%)	172 (30.7%)	171 (31.7%)	1 (4.5%)	1 (1.0%)	50 (28.2%)	50 (28.4%)	0 (0.0%)	1 (1.9%)
Jujuy	0 (0.0%)	0 (0.0%)	0 (0.0%)	0 (0.0%)	49 (8.7%)	48 (8.9%)	1 (4.5%)	11 (11.5%)	47 (26.6%)	47 (26.7%)	0 (0.0%)	12 (23.1%)
La Pampa	0 (0.0%)	0 (0.0%)	0 (0.0%)	0 (0.0%)	25 (4.5%)	20 (3.7%)	5 (22.7%)	2 (2.1%)	11 (6.2%)	11 (6.3%)	0 (0.0%)	2 (3.8%)
La Rioja	0 (0.0%)	0 (0.0%)	0 (0.0%)	0 (0.0%)	17 (3.0%)	14 (2.6%)	3 (13.6%)	3 (3.1%)	15 (8.5%)	15 (8.5%)	0 (0.0%)	4 (7.7%)
Mendoza	0 (0.0%)	0 (0.0%)	0 (0.0%)	0 (0.0%)	24 (4.3%)	18 (3.3%)	6 (27.3%)	2 (2.1%)	10 (5.6%)	9 (5.1%)	1 (100.0%)	3 (5.8%)
Misiones	0 (0.0%)	0 (0.0%)	0 (0.0%)	0 (0.0%)	335 (59.7%)	331 (61.4%)	4 (18.2%)	39 (40.6%)	63 (35.6%)	62 (35.2%)	1 (100.0%)	2 (3.8%)
Neuquén	0 (0.0%)	0 (0.0%)	0 (0.0%)	0 (0.0%)	19 (3.4%)	14 (2.6%)	5 (22.7%)	1 (1.0%)	23 (13.0%)	23 (13.1%)	0 (0.0%)	9 (17.3%)
Río Negro	0 (0.0%)	0 (0.0%)	0 (0.0%)	0 (0.0%)	24 (4.3%)	19 (3.5%)	5 (22.7%)	4 (4.2%)	24 (13.6%)	24 (13.6%)	0 (0.0%)	7 (13.5%)
Salta	0 (0.0%)	0 (0.0%)	0 (0.0%)	0 (0.0%)	160 (28.5%)	156 (28.9%)	4 (18.2%)	12 (12.5%)	54 (30.5%)	53 (30.1%)	1 (100.0%)	10 (19.2%)
San Juan	0 (0.0%)	0 (0.0%)	0 (0.0%)	0 (0.0%)	26 (4.6%)	20 (3.7%)	6 (27.3%)	6 (6.3%)	15 (8.5%)	14 (8.0%)	1 (100.0%)	5 (9.6%)
San Luis	0 (0.0%)	0 (0.0%)	0 (0.0%)	0 (0.0%)	21 (3.7%)	16 (3.0%)	5 (22.7%)	4 (4.2%)	18 (10.2%)	18 (10.2%)	0 (0.0%)	7 (13.5%)
Santa Cruz	0 (0.0%)	0 (0.0%)	0 (0.0%)	0 (0.0%)	13 (2.3%)	7 (1.3%)	6 (27.3%)	1 (1.0%)	4 (2.3%)	4 (2.3%)	0 (0.0%)	1 (1.9%)
Santa Fe	0 (0.0%)	0 (0.0%)	0 (0.0%)	0 (0.0%)	215 (38.3%)	211 (39.1%)	4 (18.2%)	2 (2.1%)	53 (29.9%)	52 (29.5%)	1 (100.0%)	0 (0.0%)
Santiago del Estero	0 (0.0%)	0 (0.0%)	0 (0.0%)	0 (0.0%)	49 (8.7%)	47 (8.7%)	2 (9.1%)	3 (3.1%)	31 (17.5%)	31 (17.6%)	0 (0.0%)	2 (3.8%)
Tierra del Fuego	0 (0.0%)	0 (0.0%)	0 (0.0%)	0 (0.0%)	8 (1.4%)	5 (0.9%)	3 (13.6%)	0 (0.0%)	0 (0.0%)	0 (0.0%)	0 (0.0%)	0 (0.0%)
Tucumán	0 (0.0%)	0 (0.0%)	0 (0.0%)	0 (0.0%)	60 (10.7%)	56 (10.4%)	4 (18.2%)	6 (6.3%)	27 (15.3%)	27 (15.3%)	0 (0.0%)	7 (13.5%)
Argentina Sea	574 (100%)	570 (99.3%)	4 (0;7%)	20 (3.5%)**	0 (0.0%)	0 (0.0%)	0 (0.0%)	0 (0.0%)	0 (0.0%)	0 (0.0%)	0 (0.0%)	0 (0.0%)

**Table A2.**

Taxonomic group	Reptiles				Birds				Mammals			
Province	Richness	Native	Exotic	Endemic	Richness	Native	Exotic	Endemic	Richness	Native	Exotic	Endemic
Buenos Aires*	60 (13.3%)	56 (12.6%)	4 (100.0%)	12 (5.6%)	479 (43.0%)	471 (42.7%)	8 (72.7%)	5 (23.8%)	122 (28.5%)	112 (27.5%)	10 (47.6%)	10 (11.5%)
Catamarca	79 (17.6%)	79 (17.7%)	0 (0.0%)	41 (19.0%)	437 (39.3%)	435 (39.5%)	2 (18.2%)	11 (52.4%)	91 (21.3%)	87 (21.4%)	4 (19.0%)	23 (26.4%)
Chaco	100 (22.2%)	99 (22.2%)	1 (25.0%)	1 (0.5%)	422 (37.9%)	419 (38.1%)	2 (18.2%)	3 (14.3%)	102 (23.8%)	96 (23.6%)	6 (28.6%)	5 (5.7%)
Chubut	53 (11.8%)	53 (11.9%)	0 (0.0%)	34 (15.7%)	283 (25.4%)	280 (25.4%)	3 (27.3%)	6 (28.6%)	98 (22.9%)	91 (22.4%)	7 (33.3%)	14 (16.1%)
Córdoba	70 (15.6%)	70 (15.7%)	0 (0.0%)	16 (7.4%)	410 (36.8%)	406 (36.8%)	4 (36.4%)	7 (33.3%)	74 (17.3%)	64 (15.7%)	10 (47.6%)	10 (11.5%)
Corrientes	104 (23.1%)	103 (23.1%)	1 (25.0%)	4 (1.9%)	511 (45.9%)	509 (46.2%)	2 (18.2%)	1 (4.8%)	108 (25.2%)	98 (24.1%)	10 (47.6%)	5 (5.7%)
Entre Ríos	63 (14.0%)	63 (14.1%)	0 (0.0%)	0 (0.0%)	385 (34.6%)	381 (34.6%)	4 (36.4%)	3 (14.3%)	64 (15.0%)	56 (13.8%)	8 (38.1%)	3 (3.4%)
Formosa	91 (20.2%)	90 (20.2%)	1 (25.0%)	2 (0.9%)	429 (38.5%)	427 (38.7%)	2 (18.2%)	2 (9.5%)	108 (25.2%)	102 (25.1%)	6 (28.6%)	3 (3.4%)
Jujuy	65 (14.4%)	65 (14.6%)	0 (0.0%)	6 (2.8%)	584 (52.5%)	582 (52.8%)	2 (18.2%)	6 (28.6%)	139 (32.5%)	135 (33.2%)	4 (19.0%)	8 (9.2%)
La Pampa	48 (10.7%)	48 (10.8%)	0 (0.0%)	20 (9.3%)	280 (25.2%)	277 (25.1%)	3 (27.3%)	6 (28.6%)	53 (12.4%)	45 (11.1%)	8 (38.1%)	10 (11.5%)
La Rioja	60 (13.3%)	60 (13.5%)	0 (0.0%)	33 (15.3%)	370 (33.2%)	368 (33.4%)	2 (18.2%)	10 (47.6%)	67 (15.7%)	63 (15.5%)	4 (19.0%)	16 (18.4%)
Mendoza	80 (17.8%)	80 (17.9%)	0 (0.0%)	47 (21.8%)	325 (29.2%)	321 (29.1%)	4 (36.4%)	9 (42.9%)	74 (17.3%)	65 (16.0%)	9 (42.9%)	21 (24.1%)
Misiones	100 (22.2%)	99 (22.2%)	1 (25.0%)	0 (0.0%)	561 (50.4%)	559 (50.7%)	2 (18.2%)	1 (4.8%)	131 (30.6%)	126 (31.0%)	5 (23.8%)	3 (3.4%)
Neuquén	69 (15.3%)	69 (15.5%)	0 (0.0%)	48 (22.2%)	268 (24.1%)	263 (23.9%)	5 (45.5%)	7 (33.3%)	69 (16.1%)	58 (14.3%)	11 (52.4%)	10 (11.5%)
Río Negro	73 (16.2%)	72 (16.1%)	1 (25.0%)	47 (21.8%)	323 (29.0%)	320 (29.0%)	3 (27.3%)	6 (28.6%)	95 (22.2%)	87 (21.4%)	8 (38.1%)	9 (10.3%)
Salta	116 (25.8%)	116 (26.0%)	0 (0.0%)	23 (10.6%)	603 (54.2%)	600 (54.4%)	3 (27.3%)	8 (38.1%)	159 (37.1%)	155 (38.1%)	4 (19.0%)	15 (17.2%)
San Juan	63 (14.0%)	63 (14.1%)	0 (0.0%)	38 (17.6%)	316 (28.4%)	314 (28.5%)	2 (18.2%)	8 (38.1%)	50 (11.7%)	46 (11.3%)	4 (19.0%)	14 (16.1%)
San Luis	50 (11.1%)	50 (11.2%)	0 (0.0%)	19 (8.8%)	319 (28.7%)	316 (28.7%)	3 (27.3%)	8 (38.1%)	51 (11.9%)	42 (10.3%)	9 (42.9%)	9 (10.3%)
Santa Cruz	31 (6.9%)	31 (7.0%)	0 (0.0%)	17 (7.9%)	252 (22.6%)	249 (22.6%)	3 (27.3%)	5 (23.8%)	82 (19.2%)	76 (18.7%)	6 (28.6%)	7 (8.0%)
Santa Fe	82 (18.2%)	81 (18.2%)	1 (25.0%)	3 (1.4%)	437 (39.3%)	434 (39.4%)	3 (27.3%)	4 (19.0%)	72 (16.8%)	62 (15.2%)	10 (47.6%)	2 (2.3%)
Santiago del Estero	68 (15.1%)	68 (15.2%)	0 (0.0%)	9 (4.2%)	376 (33.8%)	374 (33.9%)	2 (18.2%)	5 (23.8%)	74 (17.3%)	72 (17.7%)	2 (9.5%)	6 (6.9%)
Tierra del Fuego	1 (0.2%)	1 (0.2%)	0 (0.0%)	0 (0.0%)	228 (20.5%)	226 (20.5%)	2 (18.2%)	5 (23.8%)	64 (15.0%)	57 (14.0%)	7 (33.3%)	1 (1.1%)
Tucumán	68 (15.1%)	66 (14.8%)	2 (50.0%)	19 (8.8%)	500 (44.9%)	498 (45.2%)	2 (18.2%)	10 (47.6%)	112 (26.2%)	107 (26.3%)	5 (23.8%)	15 (17.2%)
Argentina Sea	3 (0.7%)	3 (0.7%)	0 (0.0%)	0 (0.0%)	64 (5.8%)	64 (5.8%)	0 (0.0%)	0 (0.0%)	52 (12.1%)	52 (12.8%)	0 (0.0%)	0 (0.0%)

**Table A3.**

Province	Total			
	Richness	Native	Exotic	Endemic
Buenos Aires*	918 (27.8%)	884 (27.3%)	34 (54.0%)	35 (7.1%)
Catamarca	671 (20.3%)	662 (20.4%)	9 (14.3%)	92 (18.7%)
Chaco	838 (25.4%)	828 (25.6%)	10 (15.9%)	13 (2.6%)
Chubut	468 (14.2%)	453 (14.0%)	15 (23.8%)	60 (12.2%)
Córdoba	643 (19.5%)	622 (19.2%)	21 (33.3%)	50 (10.2%)
Corrientes	1,079 (32.7%)	1,065 (32.9%)	14 (22.2%)	20 (4.1%)
Entre Ríos	819 (24.8%)	801 (24.7%)	16 (25.4%)	10 (2.0%)
Formosa	850 (25.7%)	840 (25.9%)	10 (15.9%)	9 (1.8%)
Jujuy	884 (26.8%)	877 (27.1%)	7 (11.1%)	43 (8.7%)
La Pampa	417 (12.6%)	401 (12.4%)	16 (25.4%)	40 (8.1%)
La Rioja	529 (16.0%)	520 (16.0%)	9 (14.3%)	66 (13.4%)
Mendoza	513 (15.5%)	493 (15.2%)	20 (31.7%)	82 (16.7%)
Misiones	1,190 (36.0%)	1177 (36.3%)	13 (20.6%)	45 (9.1%)
Neuquén	448 (13.6%)	427 (13.2%)	21 (33.3%)	75 (15.2%)
Río Negro	540 (16.3%)	523 (16.1%)	17 (27.0%)	73 (14.8%)
Salta	1,092 (33.1%)	1,080 (33.3%)	12 (19.0%)	68 (13.8%)
San Juan	470 (14.2%)	457 (14.1%)	13 (20.6%)	71 (14.4%)
San Luis	459 (13.9%)	442 (13.6%)	17 (27.0%)	47 (9.6%)
Santa Cruz	382 (11.6%)	367 (11.3%)	15 (23.8%)	31 (6.3%)
Santa Fe	859 (26.0%)	840 (25.9%)	19 (30.2%)	11 (2.2%)
Santiago del Estero	598 (18.1%)	592 (18.3%)	6 (9.5%)	25 (5.1%)
Tierra del Fuego	304 (9.2%)	292 (9.0%)	12 (19.0%)	6 (1.2%)
Tucumán	767 (23.2%)	754 (23.3%)	13 (20.6%)	57 (11.6%)
Argentina Sea	119 (3.6%)	119 (3.7%)	0 (0.0%)	20 (4.1%)

## ﻿Appendix 2

Number of exclusive endemic species. * Buenos Aires includes Ciudad Autónoma de Buenos Aires.

**Table A4.**

Province	Marine fishes	Freshwater fishes	Amphibians	Reptiles	Birds	Mammals	Total
Buenos Aires*	–	3	1	6	–	4	**14**
Catamarca	–	5	4	17	–	5	**31**
Chaco	–	2	–	–	–	1	**3**
Chubut	–	1	1	13	–	4	**19**
Córdoba	–	3	2	–	–	4	**9**
Corrientes	–	1	2	3	–	2	**8**
Entre Ríos	–	2	–	–	–	–	**2**
Formosa	–	–	–	1	–	–	**1**
Jujuy	–	3	6	2	–	2	**13**
La Pampa	–	–	–	–	–	1	**1**
La Rioja	–	2	1	7	–	3	**13**
Mendoza	–	1	1	12	–	5	**19**
Misiones	–	35	2	–	–	1	**38**
Neuquén	–	–	6	26	–	1	**33**
Río Negro	–	1	2	21	–	–	**24**
Salta	–	5	2	8	–	2	**17**
San Juan	–	5	2	9	–	3	**19**
San Luis	–	–	–	–	–	–	–
Santa Cruz	–	1	–	10	1	–	**12**
Santa Fe	–	1	–	–	–	1	**2**
Santiago del Estero	–	–	–	–	–	–	–
Tierra del Fuego	–	–	–	–	5	1	**6**
Tucumán	–	3	1	5	–	8	**17**
Argentina Sea	20	–	–	–	–	0	**20**
	**20**	**74**	**33**	**140**	**6**	**48**	**321**

## ﻿Appendix 3

Number and percentage of threatened (CR, EN, VU) species per taxonomic group and province. Percentages are calculated in relation to the total native species of the group present in the province. * Total over the number of threatened native species present in the province. ** Buenos Aires includes Ciudad Autónoma de Buenos Aires.

**Table A5.**

Province	Marine fishes	Freshwater fishes	Amphibians	Reptiles	Birds	Mammals	Total*
Buenos Aires**	0 (0.0%)	6 (2.8%)	4 (13.3%)	12 (20.0%)	56 (11.7%)	14 (11.5%)	92 (10.4%)
Catamarca	0 (0.0%)	2 (5.7%)	7 (26.9%)	12 (15.2%)	38 (8.7%)	13 (14.3%)	72 (10.9%)
Chaco	0 (0.0%)	4 (2.4%)	1 (2.1%)	24 (24.0%)	46 (10.9%)	18 (17.6%)	93 (11.2%)
Chubut	0 (0.0%)	5 (41.7%)	7 (41.2%)	9 (17.0%)	35 (12.4%)	14 (14.3%)	70 (15.5%)
Córdoba	0 (0.0%)	8 (16.0%)	3 (9.1%)	15 (21.4%)	34 (8.3%)	9 (12.2%)	69 (11.1%)
Corrientes	0 (0.0%)	5 (1.7%)	2 (3.4%)	24 (23.1%)	65 (12.7%)	17 (15.7%)	113 (10.6%)
Entre Ríos	0 (0.0%)	5 (1.9%)	3 (7.1%)	9 (14.3%)	34 (8.8%)	6 (9.4%)	57 (7.1%)
Formosa	0 (0.0%)	3 (1.8%)	1 (2.0%)	17 (18.7%)	46 (10.7%)	17 (15.7%)	84 (10.0%)
Jujuy	0 (0.0%)	4 (8.3%)	11 (23.4%)	9 (13.8%)	59 (10.1%)	24 (17.3%)	107 (12.2%)
La Pampa	0 (0.0%)	0 (0.0%)	1 (9.1%)	6 (12.5%)	23 (8.2%)	4 (7.5%)	34 (8.5%)
La Rioja	0 (0.0%)	1 (7.1%)	1 (6.7%)	10 (16.7%)	28 (7.6%)	10 (14.9%)	50 (9.6%)
Mendoza	0 (0.0%)	0 (0.0%)	1 (10.0%)	18 (22.5%)	29 (8.9%)	9 (12.2%)	57 (11.6%)
Misiones	0 (0.0%)	10 (3.0%)	1 (1.6%)	39 (39.0%)	95 (16.9%)	31 (23.7%)	176 (15.0%)
Neuquén	0 (0.0%)	4 (28.6%)	8 (34.8%)	12 (17.4%)	23 (8.6%)	11 (15.9%)	58 (13.6%)
Río Negro	0 (0.0%)	5 (26.3%)	8 (33.3%)	15 (20.5%)	39 (12.1%)	13 (13.7%)	80 (15.3%)
Salta	0 (0.0%)	9 (5.8%)	11 (20.4%)	21 (18.1%)	65 (10.8%)	25 (15.7%)	131 (12.1%)
San Juan	0 (0.0%)	1 (5.0%)	1 (6.7%)	10 (15.9%)	22 (7.0%)	8 (16.0%)	42 (9.2%)
San Luis	0 (0.0%)	2 (12.5%)	0 (0.0%)	4 (8.0%)	21 (6.6%)	6 (11.8%)	33 (7.5%)
Santa Cruz	0 (0.0%)	3 (42.9%)	1 (25.0%)	2 (6.5%)	42 (16.7%)	12 (14.6%)	60 (16.3%)
Santa Fe	0 (0.0%)	8 (3.8%)	2 (3.8%)	14 (17.1%)	43 (9.8%)	8 (11.1%)	75 (8.9%)
Santiago del Estero	0 (0.0%)	3 (6.4%)	1 (3.2%)	10 (14.7%)	31 (8.2%)	10 (13.5%)	55 (9.3%)
Tierra del Fuego	0 (0.0%)	4 (80.0%)	0 (0.0%)	0 (0.0%)	45 (19.7%)	7 (10.9%)	56 (19.2%)
Tucumán	0 (0.0%)	3 (5.4%)	6 (22.2%)	9 (13.2%)	51 (10.2%)	15 (13.4%)	84 (11.1%)
Argentina Sea	74 (13.0%)	0 (0.0%)	0 (0.0%)	3 (100%)	17 (26.6%)	8 (15.4%)	102 (14.9%)
